# Leprosy services in primary health care in India: comparative economic cost analysis of two public‐health settings

**DOI:** 10.1111/tmi.13182

**Published:** 2018-12-06

**Authors:** Anuj Tiwari, David J. Blok, Pramilesh Suryawanshi, Akash Raikwar, Mohammad Arif, Jan Hendrik Richardus

**Affiliations:** ^1^ Department of Public Health Erasmus MC University Medical Center Rotterdam Rotterdam The Netherlands; ^2^ Netherlands Leprosy Relief India Country Office New Delhi India

**Keywords:** leprosy, cost analysis, health systems, primary care, lèpre, analyse de coûts, systèmes de santé, soins primaires

## Abstract

**Objectives:**

The WHO recommends inclusion of post‐exposure chemoprophylaxis with single‐dose rifampicin in national leprosy control programmes. The objective was to estimate the cost of leprosy services at primary care level in two different public‐health settings.

**Methods:**

Ingredient‐based costing was performed in eight primary health centres (PHCs) purposively selected in the Union Territory of Dadra and Nagar Haveli (DNH) and the Umbergaon block of Valsad district, Gujarat, India. All costs were bootstrapped, and to estimate the variation in total cost under uncertainty, a univariate sensitivity analysis was performed.

**Results:**

The mean annual cost of providing leprosy services was USD 29 072 in the DNH PHC (95% CI: 22 125–36 020) and USD 11 082 in Umbergaon (95% CI: 8334–13 830). The single largest cost component was human resources: 79% in DNH and 83% in Umbergaon. The unit cost for screening the contact of a leprosy patient was USD 1 in DNH (95% CI: 0.8–1.2) and USD 0.3 in Umbergaon (95% CI: 0.2–0.4). In DNH, the unit cost of delivering single‐dose of rifampicin (SDR) as chemoprophylaxis for contacts was USD 2.9 (95% CI: 2.5–3.7).

**Conclusions:**

The setting with an enhanced public‐health financing system invests more in leprosy services than a setting with fewer financial resources. In terms of leprosy visits, the enhanced public‐health system is hardly more expensive than the non‐enhanced public‐health system. The unit cost of contact screening is not high, favouring its sustainability in the programme.

## Abstract (fr)

**Objectifs:**

L’OMS recommande l'inclusion de la chimioprophylaxie post‐exposition à la rifampicine à dose unique dans les programmes nationaux de lutte contre la lèpre. L'objectif était d'estimer le coût des services contre la lèpre au niveau des soins primaires dans deux contextes de santé publique différents.

**Méthodes:**

Le calcul des coûts en fonction des ingrédients a été effectué dans 8 centres de santé primaires (CSP) choisis à dessein dans le Territoire d'Union de Dadra et Nagar Haveli (DNH) et dans le bloc d'Umbergaon du district de Valsad, dans le Gujarat, en Inde. Tous les coûts ont été amorcés et, pour estimer la variation du coût total sous incertitude, une analyse de sensibilité univariée a été réalisée.

**Résultats:**

Le coût annuel moyen de la fourniture des services contre la lèpre était de 29.072 USD dans les CSP de DNH (IC95%: 22.125 à 36.020) et de 11.082 USD à Umbergaon (IC95%: 8.334 à 13.830). Les ressources humaines constituaient la principale composante de coût: 79% à DNH et 83% à Umbergaon. Le coût unitaire de dépistage du contact d'un patient atteint de lèpre était de 1 USD à DNH (IC95%: 0,8 à 1,2) et de 0,3 USD à Umbergaon (IC95%: 0,2 à 0,4). A DNH, le coût unitaire de l'administration d'une dose unique de rifampicine comme chimioprophylaxie pour les contacts était de 2,9 USD (IC95%: 2,5 à 3,7).

**Conclusions:**

Un cadre doté d'un système de financement renforcé pour la santé publique investit davantage dans les services de lutte contre la lèpre qu'un cadre disposant de moins de ressources financières. En ce qui concerne les visites pour la lèpre, le système de santé publique renforcé n'est guère plus coûteux que le système de santé publique non renforcé. Le coût unitaire du dépistage des contacts n'est pas élevé, ce qui favorise sa durabilité dans le programme.

## Introduction

Leprosy is a chronic infectious disease caused by *Mycobacterium leprae*. While the first outward sign is usually discoloured painless skin patches, a delay in diagnosis can lead to complications including physical disability 1. Many challenges are associated with leprosy infection. First, the transmission mechanism is unclear 2, and the incubation period with active transmission is long 3. Second, those affected are vulnerable to co‐infections and mental health problems 4, 5. Third, the stigma caused by leprosy is more severe than that of other stigma‐causing diseases such as epilepsy and tuberculosis 6; stigma not only isolates socially but also restricts employment opportunities 7, 8. Fourth, disability is reported mainly in the productive age group and interrupts employment, sometimes lifelong 9. Finally, the afflicted population is mainly poor 4, 10, and the cost of treatment imposes a high burden on households 11.

Sixty per cent of the 210 758 new leprosy cases diagnosed worldwide in 2015 were diagnosed in India 12. New case detection has remained almost stagnant in the past 9 years, indicating uninterrupted transmission 12, 13. Although the Indian National Leprosy Eradication Program (NLEP) showed an annual new case detection rate (ANCDR) of 9.71 per 100 000 and a prevalence rate of 0.66 per 10 000 population in 2015–16 13, national average rates are not representative of leprosy‐affected pockets. In 80 districts (12% of all districts in India), the ANCDR per 100 000 population was over 20 new cases, and 22 districts (3% of all districts) reported a rate higher than 50 new cases 13. After the declaration of prevalence‐based elimination in 2005, the Indian program was criticised for being passive and missing new cases 14, 15. Therefore, in 2016, the NLEP started a door‐to‐door Leprosy Case Detection Campaign (LCDC), covering 149 districts across 19 states 16, 17. Since inception, LCDC claimed to detect more than 34 000 new cases under NLEP (source: Central Leprosy Division, India), but this figure still lies below the number of estimated hidden cases 14, 15.

As leprosy recently gained a significant political commitment from the Government of India, it is now back on the agenda of the Ministry of Health and Family Welfare 18. The 2017 parliamentary budget speech also included a commitment to eradicate leprosy by 2018 19 (a target that seems unrealistic given the present epidemiological level) 15. However, as the NLEP is in the process of testing feasible strategies for interrupting transmission of *M. leprae*, economic analysis, particularly costing estimates, is important to guide the decisions that aim to improve financial efficiency 20.

As costing estimates at primary‐care level in leprosy are scarce 21, our study aimed to estimate the cost of leprosy services at primary care level in two different public‐health settings. Because health care in India is organised at the provincial level, individual public‐health settings differ in factors such as funding, staffing and infrastructure, which are linked directly to the cost of services and indirectly to service coverage. To gain an overview of the possible variation in costs, we therefore examined two different public‐health settings. The purpose of this study was to mainly provide cost estimates that can aid financial planning of a scale‐up and assessing the cost‐effectiveness of leprosy control activities, including post‐exposure prophylaxis with single‐dose of rifampicin (SDR).

## Methods

### Study sites

Data were collected in the Union Territory of Dadra and Nagar Haveli (DNH) and the adjoining Umbergaon block of Valsad district, Gujarat, each a tribal area with a similar demographic and socioeconomic structure (Table 1). A block is a district subdivision administrative unit. As each area is rated as highly endemic for leprosy, its leprosy epidemiology is also comparable 13. The public‐health systems are nonetheless different, because DNH operates directly under the federal government and receives a higher central budgetary assistance per capita for overall and health funding alike 22, 23. Relative to Umbergaon, DNH's public‐health system is enhanced in terms of its available infrastructure (Table 1), human resources (Table S1), and service delivery coverage (Table S2) 24. Due to these factors, leprosy patients’ out‐of‐pocket expenditure on primary care in DNH is lower 25.

**Table 1:** Comparison of Dadra and Nagar Haveli and Umbergaon with regard to demography, epidemiology, socioeconomic factors and public‐health facilities

Indicators	DNH	Umbergaon
Demographic and socioeconomic indicators (census 2011)		
Number of households (HH)	76 121	54 814
Population	343 709	261 204
Rural population	53.2%	68.7%
Females (per 1000 males)	774	933
Literacy	76.2%	69.5%
Schedule tribes†	51.9%	51.3%
Total working population	45.7%	40.4%
Epidemiology (2015–2016)		
Leprosy‐screened population	388 613	371 731
New cases detected	425	287
NCDR (per 100 000 per year)	109.3	77.2
New child cases (age <14 years)	23.2%	16.0%
New female cases	57.8%	61.6%
Prevalence/Registered patient rate (per 10 000 per year)	6.7	3.8
Grade II disability in new cases	3.3%	2.4%
PB/MB ratio	2.7	3.1
Public‐health Infrastructure (2015–2016)		
Area (sq. km)	491	343
Primary health centres (PHC)	15	10
Sub‐centres	50	64
Average population screened for leprosy by health centre	25 907	37 173

MB, multibacillary; NCDR, new case detection rate; PB, paucibacillary; Source: Tiwari *et al*. 2017 30.

† The Scheduled Castes (SCs) and Scheduled Tribes (STs) are officially designated groups of historically disadvantaged indigenous people in India.

In both areas, leprosy services are integrated into the local public healthcare delivery system. Besides the routine leprosy programme activities, an annual LCDC campaign has also been performed since 2016 in DNH and Umbergaon. Since March 2015, the LPEP program is ongoing in DNH, but not in Umbergaon. The DNH was selected for LPEP due to the highest new case detection rate (NCDR), child case rate and prevalence rate in the year 2014–15 26. Moreover, DNH had a better infrastructure and human resources in place to experiment a pilot project. The LPEP program—whose details are available in the published protocol 27—is intended to assess the feasibility and impact of contact tracing and administration of single‐dose rifampicin (SDR) to asymptomatic contacts of leprosy cases 27. The contact was defined as someone who has had prolonged or regular contact with an index case. The efficacy of SDR was already established 28 and found to have a protective effect of 57% 29. The operational alignment of LPEP and NLEP is described elsewhere 30. By including leprosy patients’ neighbours and social contacts, the LPEP has intensified contact tracing, improved screening sensitivity and broadened the coverage of contact examination.

**Figure 1:**
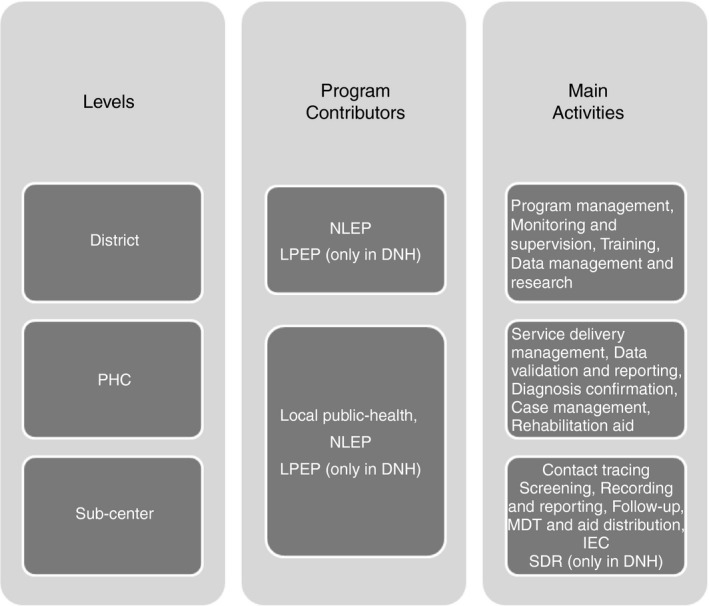
Data collection levels and corresponding leprosy control activities in DNH and Umbergaon.

### Cost data collection and analysis

When designing the study, we referred to the ‘WHO Guide to Cost‐Effectiveness Analysis’ and ‘Drummond's check‐list for assessing economic evaluations’ 31, 32, taking the perspective of the health system. The first step was to identify financial sources that contribute to leprosy service delivery: the local public‐health system, the NLEP and LPEP (donor funds for DNH only). The data related to sampled primary health centres (PHCs) were spread at three levels: district (which included only NLEP and LPEP expenditures), PHCs and sub‐centre (Figure 1). The sub‐centres, which are the most peripheral health units, are managed by paramedical staff and cater mainly for preventive care, with some curative services for minor ailments 20. We did not collect data on leprosy costs at international, national or state levels as we only focused on the primary care level.

The cost data were collected from June to October 2016, for eight PHCs (four PHCs at each study site), purposively selected on the basis of the leprosy prevalence of 2014–15, that is, a mix of low, medium and high leprosy endemicity (Table S3). The bottom–up ingredient‐based approach was applied to costing 33. Costs were categorised as follows: capital costs, including building, equipment and other consumables, that had lasted for more than 1 year; and recurrent costs, that is, staff salaries, reimbursements for leprosy schemes, monitoring, repeated training, drugs, consumables and overheads such as water and electricity bills, that had been incurred in the previous year (June 2015 to May 2016). After facility assessment (to list all assets), records were cross‐checked to determine the quantity of assets consumed with their purchasing price. For the assets, prices data were not available; we used data from other state government's pricing lists or open market rates. To record total working durations and the proportion of time allocated to leprosy services, staff were interviewed on the basis of a semi‐structured questionnaire. This proportion of time allocated to leprosy was multiplied with the corresponding remuneration to derive HR cost. The annual cost of buildings was estimated by multiplying the government rental rates by the roof‐covered surface area of the facilities. Equipment was annualised based on its useful life at a discount rate of 3% 31. Shared costs other than HR were apportioned on the bases of leprosy prevalence, that is, the number of patients on the treatment register (Table S3).

To review treatment progress, leprosy patients should ideally be examined every month 34. During analysis, patients on monthly multidrug therapy (MDT)—the standard leprosy treatment—were aggregated to an annual proxy of leprosy visits, which, as a proportion of annual general outpatient department (OPD) visits, served as leprosy‐proportion allocation (Table S4) 32. The term *proxy* is used because visits can be paid either by patients or by health staff; usually, patients visit PHCs irregularly and receive monthly services such as MDT and a general check‐up (treatment progress review) at their doorstep by field staff 25. Patients tend to visit health facilities only for more severe medical conditions. It was also the case that PHCs had data only on general OPD visits, which were not classified into leprosy‐related visits.

We surveyed one sub‐centre in each sampled PHC in DNH and Umbergaon and considered it as a standard example (Table S5). The cost of a standard example was multiplied by the number of sub‐centres under the respective PHC. The sub‐centres received medicines and consumables from their respective PHCs, which are covered under PHC costing and therefore excluded in the standard example to avoid double counting.

Using India GDP deflators, all prices were converted to the 2015 (base year) price. The annual costs were collected for the complete 2015–2016 financial year. US dollars (USD) were converted at an exchange rate of INR 67 35. Unit costs were derived by dividing the cost by the corresponding service output.

To overcome the small sample size, all costs were bootstrapped with 999 iterations to estimate robust point estimates and confidence intervals. We performed univariate sensitivity analysis with a first scenario of a 25% fluctuation in all cost components (upper and lower side of base estimate). In the second scenario, human resources and drug costs fluctuated by 80% on the lower side and by 100% on the upper side, and the rest of the components remained as in the first scenario (25% fluctuated on either side). In the third scenario, total programmatic (Local public‐health, NLEP and LPEP) contributions fluctuated 25% on either side. The fluctuation percentages were referred from other published literature, specific to primary health care setting in India 20.

The database was created in Microsoft Excel, cost analysis was conducted in SPSS 21, and the sensitivity analysis was conducted using SensIt (TreePlan) in Microsoft Excel.

## Results

### Profiling PHCs

A total of eight PHCs were sampled (four at each site), covering the leprosy‐related cost from district to sub‐centre levels. The DNH had six sub‐centres, and Umbergaon had nine sub‐centres attached per sampled PHC. In DNH, a health centre was staffed by a mean of 47 people; in Umbergaon, the mean was 26. Mean leprosy‐related staff (i.e., completely or partially engaged) was 31 in DNH and 23 in Umbergaon, including Accredited Social Health Activists (ASHAs), who are involved actively in NLEP.

The Umbergaon had to cater a higher mean catchment population (40 298) than DNH (27 237) with less staff. Conversely, mean general outpatient visits were higher in DNH (31 318) than in Umbergaon (22 021). The mean number of leprosy visits served by DNH (480) was twice as high as that served in Umbergaon (218). In 2015–2016, 53% more new cases were detected in DNH than in Umbergaon. On average, 29 042 contacts of leprosy patients were registered at a PHC in DNH (including those registered in the LPEP program); in Umbergaon, 38 475 contacts were registered. These covered contacts were close to the total catchment population, as they had been registered largely during the LCDC, which had had a high coverage (Table 2). The mean number of contacts registered per PHC under LPEP was 2500, and 114 Index cases.

**Table 2:** Profile of sampled Primary Health Centres in Dadra Nagar Haveli and Umbergaon in 2015–2016

S. no.	Characteristics (2015–2016)	DNH (*n* = 4)		Umbergaon (*n* = 4)	
		Mean	Range	Mean	Range
2A	Catchment population	27 237	20 644–30 800	40 298	33 500–47 665
2B	Human resources†	47	36–69	26	14–45
2C	Leprosy human resources‡	31	20–57	23	17–43
2D	General outpatient visits§	31 318	24 400–45 670	22 021	17 280–28 300
2E	Leprosy visits¶	480	80–800	218	95–376
2F	New cases detected	55	5–88	36	14–67
2G	Contacts registered under NLEP	26 542	20 644–30 800	38 475	32 561–44 032
2H	Contacts registered under LPEP	2500	200–4166	NA	

The mean refers to an average value per PHC. NA, not applicable.

† Human resources (medical and non‐medical staff, and active volunteers) deployed at or below PHC level. This does not include NLEP and LPEP staff.

‡ Human resources (medical and non‐medical staff, and active volunteers) engaged in leprosy services (exclusive or shared). This does not include NLEP and LPEP staff at district level.

§ The general outpatient visits are the number of visits, not persons.

¶ Leprosy visits calculated on the basis of leprosy prevalence.

### Annual costs

The mean annual cost of providing leprosy services was USD 29 072 (95% CI: 22 125–36 020) in DNH and USD 11 082 (95% CI: 8334–13 830) in Umbergaon. HR costs were the single largest component (79% in DNH and 83% in Umbergaon). The cost of drugs (including MDT and SDR) was 10% in DNH and 11% in Umbergaon and was followed by overhead costs of 8% in DNH and 4% in Umbergaon.

Table 3 breaks down the annual mean cost of leprosy services under various components. Unlike the proportional cost distributions, the cost estimates differed between the two areas. In Umbergaon, HR costs were 60% lower than in DNH, drug costs were 61% lower and overhead costs were 80% lower. In DNH, drug costs also included the rifampicin (SDR) used in the LPEP program.

**Table 3:** Annual cost of delivering leprosy services in DNH and Umbergaon at primary health centres

S. no.	Annual cost†	DNH (*n* = 4)					Umbergaon (*n* = 4)					*P* ‡
		Mean INR (USD)	%	95% CI		Mean INR per leprosy visit (USD)	Mean INR (USD)	%	95% CI		Mean INR per leprosy visit (USD)	
				Lower (USD)	Upper (USD)				Lower (USD)	Upper (USD)		
3A	Human resources	1 527 464 (22 798)	78.4	1 215 340 (18 139)	1 839 587 (27 457)	3181 (47.5)	612 748 (9145)	80.3	450 778 (6728)	744 181 (11 107)	2814 (42.0)	**0.017**
3B	Equipment	20 528 (306)	1.1	12 768 (191)	28 288 (422)	43 (0.6)	6405 (96)	1.1	3062 (46)	9748 (145)	29 (0.4)	0.089
3C	Drugs	202 834 (3027)	10.4	111 883 (1670)	280 991 (4194)	422 (6.3)	78 950 (1178)	11.9	53 361 (796)	109 833 (1639)	363 (5.4)	0.103
3D	Consumables	5167 (77)	0.3	3371 (50)	7107 (106)	11 (0.2)	2463 (37)	0.5	938 (14)	4872 (73)	11 (0.2)	0.223
3E	Building	31 827 (475)	1.6	18 867 (282)	44 834 (669)	66 (1.0)	9720 (145)	1.5	6076 (91)	13 718 (205)	45 (0.7)	0.101
3F	Overheads	160 021 (2388)	8.2	99 357 (1483)	220 684 (3294)	333 (5.0)	32 200 (481)	5.3	16 257 (243)	49 127 (733)	148 (2.2)	0.052
3G	Total	1 947 841 (29 072)	100	1 482 373 (22 125)	2 413 311 (36 020)	4056 (60.5)	742 490 (11 082)	100	558 392 (8334)	926 587 (13 830)	3410 (50.9)	**0.027**

The mean refers to an average value per PHC.

† Comprising NLEP and LPEP costs in DNH, and only NLEP cost in Umbergaon.

‡ ANOVA of Mean INR (USD).

The cost of MDT was dependent on treatment duration according to the type of leprosy (PB or MB). The number of MB cases was higher in DNH than in Umbergaon (Table 1). Of all components, expenditure on consumables was the smallest and can be considered as a part of expenditure on drugs or curative care. As capital costs, buildings and equipment together were also relatively low: 2.7% in DNH and 2.6% in Umbergaon (Table 3).

Table 4 shows the annual mean cost of leprosy programme components, at district and sub‐centre level. The local public‐health system's cost was 51% in DNH and 67% in Umbergaon, which was highest, compared to NLEP and LPEP (as other cost contributors). The NLEP expenditure was 31% in DNH and 33% in Umbergaon, while LPEP accounted for an additional 18% in DNH. The local public‐health system cost in Umbergaon was 50% less than DNH; the difference being statistically significant. The NLEP cost in Umbergaon was 59% less than in DNH (Table 4).

**Table 4:** Cost of leprosy programme components in leprosy service delivery in DNH and Umbergaon

S. no.	Annual cost	DNH (*n* = 4)					Umbergaon (*n* = 4)					*P* †
		Mean INR (USD)	%	95%CI		Mean INR per leprosy visit (USD)	Mean INR (USD)	%	95%CI		Mean INR per leprosy visit (USD)	
				Lower (USD)	Upper (USD)				Lower (USD)	Upper (USD)		
4A	Local public‐health	987 417 (14 738)	51	785 463 (11 723)	1 216 480 (18 156)	2056 (30.7)	494 719 (7384)	67	380 520 (5679)	557 880 (8327)	2272 (33.9)	**0.004**
4B	NLEP	608 817 (9087)	31	75 535 (1127)	971 646 (14 502)	1268 (18.9)	247 771 (3698)	33	102 480 (1530)	441 798 (6594)	1138 (17.0)	0.133
4C	LPEP	351 608 (5248)	18	32 060 (479)	566 808 (8460)	732 (10.9)		NA	NA			
4D	Total	1 947 841 (29 072)	100	1 482 373 (22125)	2 413 311 (36 020)	4056 (60.5)	742 490 (11 082)	100	558 392 (8334)	926 587 (13 830)	3410 (50.9)	**0.027**

Costs included IEC, training and other benefits; Leprosy visits are derived from prevalence. The ‘mean’ refers to an average value per PHC. LPEP, Leprosy Post‐exposure Prophylaxis (LPEP) program. NA, not applicable; NLEP, National Leprosy Eradication Program.

† ANOVA of Mean INR (USD).

### Unit cost

The unit cost was derived as a ratio of mean total annual cost (Table 3 or 4) and service output (Table 2) in that year (Table S4). Separately, the LPEP unit cost was derived from the LPEP program cost only. The unit cost for screening a leprosy patient's contact was USD 1 (95% CI: 0.8–1.2) in DNH (3G/(2G+2H)) and USD 0.3 (95% CI: 0.2–0.4) in Umbergaon (3G/2G). The number of contacts registered and screened in Umbergaon was 32% higher than in DNH (Table 2). The cost per new case detected and managed was USD 531 (95% CI: 486.7–575.3) in DNH and USD 312 (95% CI: 292.4–331.9) in Umbergaon (3G/2F). The unit cost per leprosy visit was USD 60.5 (95% CI: 59.5–61.6) in DNH and USD 50.9 (95% CI: 50.0–51.8) in Umbergaon (3G/2E). Under LPEP, the cost per person screened in DNH was USD 2.1(4C/2H). Of the total number of contacts screened (10 000) under LPEP, 7314 contacts received SDR (*n* = 4 PHC DNH) at a unit cost of USD 2.9 (95% CI: 2.5–3.7).

### Sensitivity analysis

Univariate sensitivity analysis was performed to estimate the difference between the expected value and the observed value of total annual cost, based on the uncertainty of a specific cost component. The scenarios were (1) 25% fluctuation in all cost components, (2) human resource and drug costs fluctuated by 80% on lower side and 100% on upper side, (3) total programmatic contributions fluctuating 25% on either side. The total annual cost of the leprosy programme was most sensitive to HR; in DNH, this was 97.2% for scenario 1 and 98.2% for scenario 2, against 98.1% for scenario 1 and 98.3% for scenario 2 in Umbergaon. In scenario 1, a fluctuation of 25% in HR cost resulted in 19.2% and 20.6% fluctuation in the total cost of DNH and Umbergaon, respectively. In scenario 2, a reduction in 80% in HR cost resulted in 62.7% and 66% reduction in the total cost of DNH and Umbergaon. Furthermore, a 100% increase in HR cost resulted in 78.4% and 82.5% increase in the total cost of DNH and Umbergaon.

Drugs were the next most influential component for total cost (DNH: 1.7% for scenario 1 and 2; Umbergaon: 1.6% for scenario 1 and 2). There was little variation in the percentage between the two scenarios for HR and drugs in both areas, meaning that the total cost had a low threshold level with respect to HR and the change in the cost of drugs. Changing the cost of building, equipment and consumables had a negligible impact on the total cost. In scenario 3, the total cost was most sensitive to the local public‐health system cost (66.4% in DNH and 79.9% in Umbergaon), followed by NLEP (25.2% in DNH and 20.1% in Umbergaon). A fluctuation of 25% in the local public‐health system and NLEP cost resulted into fluctuation of 12.7% and 7.8% in the total cost of DNH, respectively, and 16.7% and 8.3% in Umbergaon. The LPEP total cost for DNH had a swing of 8.4% with respect to the induced variation (Figures S1 and S2). Changing the LPEP cost by 25% resulted into a fluctuation of 4.5% in the total cost.

## Discussion

By quantifying expenditures, this study provides a detailed cost analysis of leprosy primary care in two different public‐health settings in India. The results inform about the allocative efficiency which is important for policy planning, aiming at the improvement of the leprosy control programme. As leprosy is a chronic disease whose treatment duration ranges from 6 to 12 months, primary care is an important aspect of disease management. PHCs are the nodal points for public‐health care and managing programmes at the grass‐roots level.

Indian leprosy services are now largely integrated into the general public‐health system 30. Previously, the country's National Leprosy Eradication Program (NLEP) was fully vertical, providing separate human resources and infrastructure to leprosy services, which later merged into the general health care. Nonetheless, NLEP still provides limited vertical support, especially to highly endemic areas, mainly for the following: non‐capital expenditure on diagnostics; disability (rehabilitation, reconstructive surgeries and prosthetics); Information Education and Communications (IEC); additional human resources; and research. It is also the case that a network of non‐governmental organisations (NGOs) supports various activities in line with the NLEP, including the implementation of pilot projects. The three main financial contributors in leprosy elimination are therefore the local public‐health system, NLEP and NGOs. At district level, these financial contributors have their programme management teams which support local public‐health system (PHCs and sub‐centres) to provide leprosy services.

Although the two neighbouring study areas are comparable with regard to demographic and socioeconomic factors, DNH had better resources and detected more leprosy cases than Umbergaon. The number of new cases depends not only on endemicity levels but also on active case‐finding inputs, which are resource intensive. DNH is more active in case finding because it has an additional research project, LPEP, which facilitates active case detection and contact examination. However, LCDC also contributes to new case detection (in both areas), but the contact examination is more thorough in LPEP because it requires ineligible individuals to be excluded from taking single‐dose rifampicin. Furthermore, the leprosy case‐detection campaigns (LCDC) are implemented in a periodic and rapid (campaign) mode, reaching almost the full population within a short period (1–2 months per campaign), whereas LPEP is a continuous 3‐year project. As a result, LCDC covers a higher number of contacts than LPEP.

The mean annual cost of providing leprosy services was USD 29 072 in DNH and USD 11 082 in Umbergaon. The higher PHC cost in DNH was due mainly to the additional cost of LPEP and to a higher proportion allocation (Table S4). The higher HR cost in DNH was mainly due to the higher time spent on leprosy (reported) by staff and also higher remuneration scale. However, when accounted for output (leprosy visit), the percentage difference between the costs of DNH and Umbergaon fell dramatically. The percentage difference in the total mean costs was 90%, whereas mean costs per leprosy visit were only 17% different between DNH and Umbergaon (Table 3). This means that the higher cost in DNH was related to more productivity. Next, the total cost of the NLEP was low in Umbergaon because it is a block whose resources are shared with rest of the Valsad district. The percentage difference in the mean NLEP costs was 84%, whereas NLEP costs per leprosy visit were only 10.6% different between DNH and Umbergaon. Interestingly, the local public‐health cost per leprosy visit was cheaper by USD 3.2 in DNH than Umbergaon. As exclusive public contribution, the aggregated unit cost (per visit) of Local public‐health and NLEP was still USD 1.2 cheaper in DNH than Umbergaon. Certainly, in the short run, LPEP masked the savings in DNH, but as an investment in prevention, it can still be cost‐effective in the long run (Table S6). Moreover, DNH investment in active case finding leads to early detection and prevention of new cases. In future, this will also save cost (opportunity) of other government programmes such as poverty eradication, malnutrition and disability support. Additionally, out‐of‐pocket expenditure on leprosy by households will also be saved in the long run. Our study only focused on the health system cost, but we recommend exploring opportunity costs, as there are not many data available, and required to measure the economic burden of leprosy. In both DNH (79%) and Umbergaon (83%), HR was the highest cost element. These high HR costs are in line with similar studies 20. Capital cost, in contrast, turned out to be one of the lowest cost elements: 2.7% in DNH and 2.6% in Umbergaon. This high HR cost is due to the fact that national programme at primary care level is a field‐driven public‐health programme that provides services close to the community. The implementation of such programmes needs higher investment in HR rather than in fixed infrastructure (Table S7). Patients also prefer to visit health facilities only for essential curative care such as acute co‐morbidity or leprosy reactions, and remain non‐regular for routine health check‐ups. The afflicted population is mainly poor, and indirect costs such as wage loss and transportation are a disincentive for them to pay health‐centre visits 25. The same study informed that the out‐of‐pocket expenditure due to leprosy was lower in DNH than Umbergaon 25. If aligned with our study results, then we can infer that an enhanced health system is comparatively costly, particularly due to the investment in prevention, but that it also reduces the out‐of‐pocket expenditure burden on the households.

At micro level, we also observed that the low endemic PHCs are not necessarily relatively cheap, mainly due to fixed costs such as building and equipment, and partially due to monthly recurrent salary cost.

The local public‐health system is the backbone of leprosy service delivery. The cost of local public‐health system was the highest (51% in DNH and 67% in Umbergaon), followed by NLEP and LPEP. Next, the unit costs can be used to estimate the budget by applying it to the desired level of coverage, but they are not the indicator for efficiency, therefore should not be interpreted as cost‐effectiveness of programmes. Moreover, all unit costs are derivatives of the same overall programme cost and coverage (process indicators). A more realistic approach to determining financial efficiency would be to compare costs with impact indicators using an appropriate time horizon 31. Usually, when an infectious disease programme approaches elimination, it becomes more resource intensive, but, if it possible to eliminate or eradicate the disease, is still considered worthwhile 36.

Our results can provide a basis for budgeting and financing. Due to the increased political commitment, the funds allocation for PEP should be not a problem, but timely flow of funds is a possible financial issue. More budget for HR means new recruitment, which is a lengthy process due to governmental regulations. This can lead to under spending in the initial years and adversely affect the prospective budgets for PEP. More HR also means more training; therefore, the general training budgets should also be revised accordingly. In general, there is a shortage of cost evidence on leprosy elimination for policy and planning 21. To the best of our knowledge, this is the first comprehensive costing study to take a public‐health‐system perspective on leprosy primary care in India. Two of only three studies to present some costing results were based on hospitalisation and not on primary care. The third study, which focused on leprosy case‐detection methods in Nigeria 37, was relevant, but, unlike our study, also included hospital costs. Another cost‐effectiveness study from Bangladesh presented MDT treatment programme costs, with and without post‐exposure prophylaxis (SDR) 38. Although—in line with our results—both the Nigerian and Bangladeshi studies reported HR cost as the largest component, the actual costs were not comparable due to the differences in epidemiology, unit of analysis and scope of the study. Another difference is that our study focused on primary care, whereas the other two were designed to evaluate specific activities of leprosy control. An another costing study on the PHCs (all diseases) of North Indian states, also mentioned HR cost as the single largest component 20.

A limitation of our method is that our purposive selection of PHCs to assure representation of low‐ and high‐performing PHCs may lead to a selection bias thus may not be representative of the actual distribution of low‐, medium‐ and high‐performing PHC in the areas. This, in turn, may lead to a deviation in cost estimates if extrapolated to full district/Union Territory or province. The cost difference between the two public‐health settings indicates further cost variation within India as a whole. The random sampling would either not be a suitable approach due to very small number of PHCs in the selected areas. The effect of small sample size of PHCs was minimised by the bootstrapping method. As another limitation, HR time allocation was based on self‐reporting rather than on observation, which would have been a better approach. Next, the NLEP and LPEP cost data were only available for full district level. To break down the NLEP and LPEP costs for sampled PHCs, we used the unit cost per new cases on the assumption that new case detection solely depends on active case‐finding efforts and pumped resources. In reality, new case detection also depends on epidemiology, socioeconomic and environmental factors. However, as the two areas were close with regard to epidemiologic and socioeconomic characteristics, we believe that this risk was minimal in our study. The environmental factors were also fairly similar: the two areas adjoin, are both very small and have no great geographical differences. As a strength, we used an appropriate costing method and our collection of primary data for a complete year to minimise any seasonal variations.

We conclude that, due to differences in public‐health system financing and structure, the annual leprosy cost at primary care level varies even between areas of comparable epidemiology. Our study shows that a setting with an enhanced public‐health financing system invests more in leprosy services and prevention than one with fewer financial resources. The enhanced public‐health system overall appears costly, but in terms of productivity, it no longer remains expensive. Additionally, it also facilitates reduction in out‐of‐pocket expenditure among households. Therefore, we recommend investment in the health system for prevention and increased access to services, which will promote early detection and transmission interruption. According to public‐health norms in India, more resources are needed to cover the population at risk, therefore these costs should also be seen as an input that will strengthen the overall health system.

Both systems invested mainly in human resources. In both the areas, the investment in human resources translates into active outreach programmes, particularly contact screening. The high investment in HR is essential to follow the global WHO leprosy guidelines sincerely 39.

We found that post‐exposure prophylaxis as addition to the control programme is resource intensive. However, once post‐exposure prophylaxis has been implemented in a routine setting, the costs are expected to fall. The WHO has recently recommended to use SDR for leprosy prevention, which will trigger scale‐up of post‐exposure prophylaxis 39. Our results can immediately guide the fiscal planning during scale‐up in India, and SDR role out in other countries after considering the local economies. The relatively low unit cost of contact screening favours its sustainability in the programme; however, this does not mean that contact tracing should be avoided even if costly. In general, leprosy work is facing financial constraint since the global declaration of leprosy elimination. These results are promising for advocacy and fundraising, especially in support of SDR. The unit costs are of much interest for funding agencies to reimburse on case bases and to plan a flexible investment with a measurable value of return.

## Ethics approval and consent to participate

The study was conducted under the Leprosy Post Exposure Prophylaxis (LPEP) program and was approved by the Institutional Human Ethics Committees of the National Institute of Epidemiology, India (NIE/IHEC201407‐01). In addition, approval for the study was given by the relevant national, state and district level health departments.

## Availability of data and materials

All data generated or analysed during this study are included in this published article [and its Supporting Information files].

## Funding

The study was funded by Novartis Foundation, Basel, Switzerland. The funding body played no role in the design of the study, collection, analysis, and interpretation of data or writing the manuscript.

## Supporting information


**Figure S1.** Tornado diagram (sensitivity analysis) for DNH leprosy services in primary care.

**Figure S2.** Tornado diagram (sensitivity analysis) for Umbergaon leprosy services in primary care.

**Table S1.** Table. Human Resources in position (permanent and contractual) in DNH and Umbergaon in 2016–17 (all UT/district).
**Table S2.** Service delivery coverage of general public‐health system in DNH and Umbergaon in 2014–15.
**Table S3.** Leprosy epidemiological profile of the PHCs sampled in DNH and Umbergaon.
**Table S4.** Cost and service delivery performance of the PHCs sampled in DNH and Umbergaon.
**Table S5.** Sub‐centre standard case: resource parameters.
**Table S6.** LPEP annual implementation performance in DNH 2015–2016 (all PHCs).
**Table S7.** Annual cost categorisation.

## References

[1] Suzuki K , Akama T , Kawashima A , Yoshihara A , Yotsu RR , Ishii N . Current status of leprosy: epidemiology, basic science and clinical perspectives. J Dermatol 2012: 39: 121–129. 21973237 10.1111/j.1346-8138.2011.01370.x

[2] Bratschi MW , Steinmann P , Wickenden A , Gillis TP . Current knowledge on Mycobacterium leprae transmission: a systematic literature review. Lepr Rev 2015: 86: 142–155. 26502685

[3] Walker SL , Lockwood DN . The clinical and immunological features of leprosy. Br Med Bull 2006: 77–78: 103–121. 10.1093/bmb/ldl010 17090777

[4] Feenstra SG , Nahar Q , Pahan D , Oskam L , Richardus JH . Recent food shortage is associated with leprosy disease in Bangladesh: a case‐control study. PLoS Negl Trop Dis 2011: 5: e1029. 21572979 10.1371/journal.pntd.0001029 PMC3091833

[5] Rocha‐Leite CI , Borges‐Oliveira R , Araujo‐de‐Freitas L , Machado PR , Quarantini LC . Mental disorders in leprosy: an underdiagnosed and untreated population. J Psychosom Res 2014: 76: 422–425. 24745785 10.1016/j.jpsychores.2014.02.006

[6] Tsutsumi A , Izutsu T , Islam AM , Maksuda AN , Kato H , Wakai S . The quality of life, mental health, and perceived stigma of leprosy patients in Bangladesh. Soc Sci Med 2007: 64: 2443–2453. 17382441 10.1016/j.socscimed.2007.02.014

[7] Rao PS , Raju MS , Barkataki A , Nanda NK , Kumar S . Extent and correlates of leprosy stigma in rural India. Indian J Lepr 2008: 80: 167–174. 19425512

[8] Ebenso B , Ayuba M . ‘Money is the vehicle of interaction’: insight into social integration of people affected by leprosy in northern Nigeria. Lepr Rev 2010: 81: 99–110. 20825114

[9] Rao PS , Darlong F , Timothy M , Kumar S , Abraham S , Kurian R . Disability adjusted working life years (DAWLYs) of leprosy affected persons in India. Indian J Med Res 2013: 137: 907–910. 23760375 PMC3734681

[10] Kerr‐Pontes LR , Barreto ML , Evangelista CM , Rodrigues LC , Heukelbach J , Feldmeier H . Socioeconomic, environmental, and behavioural risk factors for leprosy in North‐east Brazil: results of a case‐control study. Int J Epidemiol 2006: 35: 994–1000. 16645029 10.1093/ije/dyl072

[11] Chandler DJ , Hansen KS , Mahato B , Darlong J , John A , Lockwood DN . Household costs of leprosy reactions (ENL) in rural India. PLoS Negl Trop Dis 2015: 9: e0003431. 25590638 10.1371/journal.pntd.0003431 PMC4295874

[12] WHO . Global leprosy update, 2015: time for action, accountability and inclusion. Wkly Epidemiol Rec 2016: 91: 405–420. 27592500

[13] CLD . NLEP Annual Report: 2015‐16. (Available from: http://nlep.nic.in/pdf/revised%20annual%20report%2031st%20March%202015-16.pdf) [15 March 2017]

[14] Smith WC , van Brakel W , Gillis T , Saunderson P , Richardus JH . The missing millions: a threat to the elimination of leprosy. PLoS Negl Trop Dis 2015: 9: e0003658. 25905706 10.1371/journal.pntd.0003658 PMC4408099

[15] Blok DJ , De Vlas SJ , Richardus JH . Global elimination of leprosy by 2020: are we on track? Parasit Vectors 2015: 8: 548. 26490878 10.1186/s13071-015-1143-4 PMC4618543

[16] CLD . Operational guidelines for leprosy case detection campaing. (Available from: http://nlep.nic.in/pdf/Final_OG_LCDC%20(1).pdf) [3 April 2017]

[17] WHO . India's massive leprosy case detection campaign. (Available from: http://www.who.int/neglected_diseases/news/India_massive_leprosy_case_detection_campaign_reaches_320_mill/en/) [3 April 2017]

[18] GoI . PM's Message on the Occasion of Anti Leprosy Day. (Available from: http://www.pmindia.gov.in/en/news_updates/pms-message-on-the-occasion-of-anti-leprosy-day/) [3 April 2017]

[19] Indianexpress . Health Budget: Reality Check. (Available from: http://indianexpress.com/article/opinion/columns/union-budget-health-budget-public-health-investment-diseases-india-rural-india-free-treatment-4509564/) [3 April 2017]

[20] Prinja S , Gupta A , Verma R *et al* Cost of delivering health care services in public sector primary and community health centres in North India. PLoS ONE 2016: 11: e0160986. 27536781 10.1371/journal.pone.0160986 PMC4990301

[21] Tiwari A , Richardus JH . Investment case concepts in leprosy elimination: a systematic review. Lepr Rev 2016: 87: 2–22. 27255054

[22] Planning Commission GoI . Annual Plan 2014‐15 [Regular Budget]. (Available from: http://planningcommission.nic.in/plans/annualplan/ann_plan2014_15.pdf) [19 April 2017]

[23] National Institute of Public Finance and Policy MoF, Governmnet of India . Health Expenditure by the Central Government in India: State level Distribution. (Available from: http://www.nipfp.org.in/media/medialibrary/2013/08/nipfp-report100911_1.pdf) [19 April 2017]

[24] MoHFW. Rural Health Statistics 2015‐16. (Available from: https://nrhm-mis.nic.in/Pages/RHS2016.aspx?RootFolder=%2FRURAL%20HEALTH%20STATISTICS%2F%28A%29RHS%20-%202016&FolderCTID=0x01200057278FD1EC909F429B03E86C7A7C3F31&View={3EF44ABD-FC77-4A1F-9195-D34FCD06C7BA) [19 April 2017]

[25] Tiwari A , Suryawanshi P , Raikwar A , Arif M , Richardus JH . Household expenditure on leprosy outpatient services in the Indian health system: a comparative study. PLoS Negl Trop Dis 2018: 12: e0006181. 29300747 10.1371/journal.pntd.0006181 PMC5771634

[26] CLD . NLEP Annual Report: 2014‐15. (Available from: http://nlep.nic.in/pdf/Progress%20report%2031st%20March%202014-15%20-.pdf) [11 May 2018]

[27] Barth‐Jaeggi T , Steinmann P , Mieras L *et al* Leprosy Post‐Exposure Prophylaxis (LPEP) programme: study protocol for evaluating the feasibility and impact on case detection rates of contact tracing and single dose rifampicin. BMJ Open 2016: 6: e013633. 10.1136/bmjopen-2016-013633 PMC5128948 27856484

[28] Moet FJ , Pahan D , Oskam L , Richardus JH , Group CS . Effectiveness of single dose rifampicin in preventing leprosy in close contacts of patients with newly diagnosed leprosy: cluster randomised controlled trial. BMJ 2008: 336: 761–764. 18332051 10.1136/bmj.39500.885752.BE PMC2287265

[29] Feenstra SG , Pahan D , Moet FJ , Oskam L , Richardus JH . Patient‐related factors predicting the effectiveness of rifampicin chemoprophylaxis in contacts: 6 year follow up of the COLEP cohort in Bangladesh. Lepr Rev 2012: 83: 292–304. 23356030

[30] Tiwari A , Mieras L , Dhakal K *et al* Introducing leprosy post‐exposure prophylaxis into the health systems of India, Nepal and Indonesia: a case study. BMC Health Serv Res 2017: 17: 684. 28962564 10.1186/s12913-017-2611-7 PMC5622547

[31] Edejer TT‐T , Baltussen R , Adam T *et al* WHO guide to Cost‐Effectiveness Analysis. (Available from:http://www.who.int/choice/publications/p_2003_generalised_cea.pdf) [3 April 2017]

[32] Drummond MF , O'Brien B , Stoddart GL , Torrance GW . Methods for the Economic Evaluation of Health Care Programmes, 3rd edn Oxford University Press: Oxford, 1999.

[33] Chapko MK , Liu CF , Perkins M , Li YF , Fortney JC , Maciejewski ML . Equivalence of two healthcare costing methods: bottom‐up and top‐down. Health Econ 2009: 18: 1188–1201. 19097041 10.1002/hec.1422

[34] CLD . National Leprosy Eradication Program: Training Manual for Medical Officers. (Available from: http://nlep.nic.in/pdf/MO%20training%20Manual.pdf) [26 September 2017]

[35] X‐Rates . USD to INR exchange rate 2016. (Available from:http://www.x-rates.com/average/?from=USD&to=INR&amount=1&year=2016). [26 September 2017]

[36] Sicuri E , Evans DB , Tediosi F . Can economic analysis contribute to disease elimination and eradication? A systematic review PLoS ONE 2015: 10: e0130603. 26070135 10.1371/journal.pone.0130603 PMC4466479

[37] Ezenduka C , Post E , John S , Suraj A , Namadi A , Onwujekwe O . Cost‐effectiveness analysis of three leprosy case detection methods in Northern Nigeria. PLoS Negl Trop Dis 2012: 6: e1818. 23029580 10.1371/journal.pntd.0001818 PMC3447964

[38] Idema WJ , Majer IM , Pahan D , Oskam L , Polinder S , Richardus JH . Cost‐effectiveness of a chemoprophylactic intervention with single dose rifampicin in contacts of new leprosy patients. PLoS Negl Trop Dis 2010: 4: e874. 21072235 10.1371/journal.pntd.0000874 PMC2970532

[39] WHO . Guidelines for the Diagnosis, Treatment and Prevention of Leprosy: Executive Summary. (Available from: http://www.searo.who.int/entity/global_leprosy_programme/approved-guidelines-leprosy-executives-summary.pdf?ua=1) [10 May 2018]

