# Supplementary material for: Leprosy services in primary health care in India: comparative economic cost analysis of two public‐health settings
Source: Trop Med Int Health. 2018 Dec 6;24(2):155–65. doi: 10.1111/tmi.13182 (PMC7379621; doi:10.1111/tmi.13182)
Supplement: Supplementary file 1 — Figure S1. Tornado diagram (sensitivity analysis) for DNH leprosy services in primary care. [file TMI-24-155-s001.docx]

**S1 Figure: Tornado diagram (sensitivity analysis) for DNH leprosy services in primary care**

Note: All figures are in INR, and mean costs (n=4) from tables 3 and 4 were used for sensitivity analysis.

*Cost components DNH*

Scenario 1: 25% fluctuation in all cost components (upper and lower side of base estimate)

Scenario 2: Only human resource and drug costs were fluctuated by 80% on lower side and 100% on

upper side

*Programatic components DNH*

Scenario 3: Local public health, NLEP and LPEP costs fluctuated 25% on either side

HS cost: Local public health system; NLEP: National Leprosy Eradication Program; LPEP: Leprosy Post-exposure prophylaxis program

All analyses were conducted using SensIt 1.53, TreePlan.com
