# Supplementary material for: Leprosy services in primary health care in India: comparative economic cost analysis of two public‐health settings
Source: Trop Med Int Health. 2018 Dec 6;24(2):155–65. doi: 10.1111/tmi.13182 (PMC7379621; doi:10.1111/tmi.13182)
Supplement: Supplementary file 3 — Table S1. Table. Human Resources in position (permanent and contractual) in DNH and Umbergaon in 2016–17 (all UT/district). Table S2. Service delivery coverage of general public‐health system in DNH and Umbergaon in 2014–15. Table S3. Leprosy epidemiological profile of the PHCs sampled in DNH and Umbergaon. Table S4. Cost and service delivery performance of the PHCs sampled in DNH and Umbergaon. Table S5. Sub‐centre standard case: resource parameters. Table S6. LPEP annual implementation performance in DNH 2015–2016 (all PHCs). Table S7. Annual cost categorisation. [file TMI-24-155-s003.docx]

| **Additional file Table 1. Human Resources in position (permanent and contractual) in DNH and Umbergaon in 2016-17 (all UT/district)** | | |
| --- | --- | --- |
| **HR status** | **DNH** | **Umbergaon** |
| ANMs | 90 | 57 |
| Staff Nurse | 230 | 14 |
| Medical Officers | 43 | 9 |
| LTs | 35 | 8 |
| Obstetricians/ Gyn | 5 | 0 |
| Pediatricians | 6 | 0 |
| Anesthetists | 4 | 0 |
| Other Specialist | 12 | 0 |
| Pharmacists | 28 | 9 |
| Radiographer | 6 | 0 |
| AYUSH MO | 11 | 4 |
| Dentists / Dental Surgeon | 5 | 0 |
| **Others** |  |  |
| OT technicians | 27 | 0 |
| Physiotherapists | 2 | 0 |
| Dietician/Nutritionist | 2 | 0 |
| Audiologist/Speech therapist | 2 | 0 |
| Optometrist/optician | 4 | 0 |
| Counsellors | 11 | 2 |
| Source: District Health Offices of DNH and Umbergaon | | |

| **Additional file Table 2. Service delivery coverage of general public-health system in DNH and Umbergaon in 2014-15** | | |
| --- | --- | --- |
| **Service Components** | **DNH** | **Umbergaon** |
| ***Maternal health*** | *Units of services delivered* | |
| Total number of pregnant women registered for ANC - 2014-15 | 16,015 | 3,885 |
| Number of pregnant women registered within first trimester - 2014-15 | 7,625 | 3,544 |
| Number of women registered under JSY - 2014-15 | 3,565 | 1,019 |
| Number of pregnant women received 3 ANC check ups - 2014-15 | 7,691 | 3,808 |
| TT2 or booster given to pregnant women (numbers) - 2014-15 | 7,678 | 3,957 |
| Number of pregnant women given 100 IFA tablets - 2014-15 | 4,246 | 4,077 |
| Number having severe anaemia (Hb<7) treated at institution - 2014-15 | 1,199 | 0 |
| Number of home deliveries attended by SBA trained (Doctor/Nurse/ANM) - 2014-15 | 9 | 0 |
| Number of home deliveries attended by Non SBA trained (trained TB/Dai) - 2014-15 | 86 | 0 |
| Deliveries conducted at public institutions - 2014-15 | 6,359 | 1,025 |
| Institutional deliveries (Public Insti,+Pvt, Insti,) - 2014-15 | 7,493 | 3,593 |
| Total reported deliveries - 2014-15 | 7,588 | 3,593 |
| Number of C-section deliveries conducted at public facilities - 2014-15 | 1,225 | 17 |
| ***Child health*** |  |  |
| Number of Infants given OPV 0 (Birth Dose) - 2014-15 | 6,379 | 3,476 |
| Number of Infants given BCG - 2014-15 | 7,918 | 3,503 |
| Number of Infants given DPT(1,2,3) - 2014-15 | 21,403 | NA |
| Number of Infants given DPT1 - 2014-15 | 6,962 | 0 |
| Number of Infants given DPT2 - 2014-15 | 7,053 | 0 |
| Number of Infants given DPT3 - 2014-15 | 7,388 | 0 |
| Number of infants given Pentavalent (1,2,3)- 2014-15 | NA | 9,605 |
| Number of Infants given Pentavalent 1 - 2014-15 | 0 | 3,148 |
| Number of Infants given Pentavalent 2 - 2014-15 | 0 | 3,130 |
| Number of Infants given Pentavalent 3 - 2014-15 | 0 | 3,327 |
| Number of Infants given Measles - 2014-15 | 7,677 | 3,378 |
| Number of fully immunized children (9-11 months) - 2014-15 | 7,473 | 3,387 |
| Vitamin - A dose 1 - 2014-15 | 4,408 | 3,340 |
| ***General public health care*** |  |  |
| IPD (Number) - 2014-15 | 68,047 | 9,897 |
| OPD (Number) - 2014-15 | 1,057,800 | 90,024 |
| Number of Major Operations - 2014-15 | 5,030 | 539 |
| Number of Minor Operations - 2014-15 | 13,488 | 198 |
| Number of Hysterectomy Surgeries - 2014-15 | 152 | 0 |
| Ayush OPD (Number) - 2014-15 | 20,531 | 12 |
| Dental OPD (Number) - 2014-15 | 45,784 | 0 |
| Adolescent counselled (Number) - 2014-15 | 307 | 4 |
| **Data sources:** |  |  |
| Performance of Key HMIS Indicators (Across Sub Districts) for Dadra and Nagar Haveli Financial Year: 2014-15 | | |
| <https://data.gov.in/resources/performance-key-hmis-indicators-across-sub-districts-dadra-and-nagar-haveli-financial-ye-0/download> | | |
| Performance of Key HMIS Indicators (Across Sub Districts) for Valsad (Gujarat) Financial Year: 2014-15 | | |
| <https://data.gov.in/resources/performance-key-hmis-indicators-across-sub-districts-valsad-gujarat-financial-year-2014-15/download> | | |

| **Additional file Table 3. Leprosy epidemiological profile of the PHCs sampled in DNH and Umbergaon** | | | | | | | | | | | | | | | | |
| --- | --- | --- | --- | --- | --- | --- | --- | --- | --- | --- | --- | --- | --- | --- | --- | --- |
| **Leprosy Profile** | **DNH PHC** | | | | | | | | **Umbergaon, Valsad PHC** | | | | | | | |
|  | **Amboli** | | **Dadra** | | **Dapada** | | **Kilavani** | | **Fansa** | | **Sanjan** | | **Dehri** | | **Valvada** | |
|  | **2014-15** | **2015-16** | **2014-15** | **2015-16** | **2014-15** | **2015-16** | **2014-15** | **2015-16** | **2014-15** | **2015-16** | **2014-15** | **2015-16** | **2014-15** | **2015-16** | **2014-15** | **2015-16** |
| Aprox. catchment Population | 58,317 | 30,800 | 59,506 | 20,644 | NA | 27,903 | 93,307 | 29,600 | 51,003 | 45,385 | 34,419 | 34,643 | 10,272 | 47,665 | 33,284 | 33,500 |
| Leprosy prevalence per 10,000 | 9.77 | 14.29 | 0.34 | 0.97 |  | 11.83 | 2.68 | 16.03 | 2.74 | 6.83 | 4.07 | 7.22 | 5.84 | 1.68 | 3.00 | 2.69 |
| New leprosy cases detected | 107 | 88 | 5 | 5 |  | 58 | 47 | 68 | 61 | 67 | 33 | 43 | 12 | 14 | 23 | 18 |
| Children among new cases (%) | 22.4 | 27.3 | 40 | 20 |  | 27.5 | 38.3 | 30.9 | 18.0 | 22.3 | 33.3 | 23.2 | 16.6 | 28.5 | 4.3 | 5.5 |
| Female among new cases (%) | 60.7 | 64.8 | 40.0 | 60.0 |  | 51.7 | 48.9 | 60.3 | 72.1 | 67.1 | 63.6 | 65.1 | 41.6 | 57.1 | 78.2 | 61.1 |
| Grade 2 disability (%) | 1.9 | 2.3 | 0.0 | 0.0 |  | 1.7 | 0.0 | 1.5 | 1.6 | 2.9 | 0.0 | 6.9 | 0.0 | 0.0 | 0.0 | 0.0 |
| MB in New Cases (%) | 26.2 | 19.3 | 40.0 | 40.0 |  | 22.4 | 29.8 | 30.9 | 13.1 | 11.9 | 21.2 | 23.2 | 33.3 | 14.2 | 26.0 | 27.7 |
| ANCDR per 100,000 population | 183.5 | 285.7 | 8.4 | 24.2 |  | 207.8 | 50.4 | 253.5 | 119.6 | 147.6 | 95.8 | 124.1 | 116.8 | 29.3 | 69.1 | 53.7 |
| Deaths due to Leprosy | 0 | 0 | 0 | 0 |  | 0 | 0 | 0 | 0 | 0 | 0 | 0 | 0 | 0 | 0 | 0 |
| NA: Not applicable as Dadra was a part of Kilavani in 2014-15  Note: The drastic difference in prevalence and NCD in DNH between 2014-16 is due to division of population by upgrading 5 health centers into PHCs | | | | | | | | | | | | | | | | |

**Additional file Table 4. Cost and service delivery performance of the PHCs sampled in DNH and Umbergaon.**

| **Service Delivery** | **DNH PHC** | | | | **Umbergaon** | | | |
| --- | --- | --- | --- | --- | --- | --- | --- | --- |
|  | **Amboli** | **Dadra** | **Dapada** | **Kilavani** | **Fansa** | **Sanjan** | **Dehri** | **Valvada** |
|  | **2015-16** | **2015-16** | **2015-16** | **2015-16** | **2015-16** | **2015-16** | **2015-16** | **2015-16** |
| Cost (USD) | 41118.4 | 13329.2 | 28038.1 | 33803.3 | 14920.6 | 12738.8 | 6375.0 | 10293.5 |
| Number of general OPD | 28,800 | 45,670 | 24,400 | 26,400 | 28,300 | 21,204 | 17,280 | 21,300 |
| Proxy leprosy visits* | 800 | 80 | 415 | 626 | 376 | 266 | 95 | 134 |
| Proportion allocation** | 0.027 | 0.002 | 0.017 | 0.023 | 0.013 | 0.012 | 0.005 | 0.006 |
| IPD | 1,500 | 25 | 20 | 2,460 | 300 | 1,488 | 20 | 360 |
| Laboratory | 6,600 | 1,225 | 2,890 | 6,240 | 3,000 | 12,816 | 600 | 10,440 |
| Persons screened under NLEP | 30,800 | 20,644 | 27,903 | 26,822 | 44,032 | 34,477 | 42,831 | 32,561 |
| Persons screened under LPEP | 4,166 | 200 | 3,006 | 2,627 | NA | NA | NA | NA |
| Contacts received SDR | 3,103 | 162 | 2,484 | 1,565 |  |  |  |  |

NA: Not applicable as no LPEP in Umbergaon

*Proxy leprosy visits (either by patient or health worker) based on the monthly prevalence of leprosy patients with an assumption of 1 visit per patient per month

** Proportion allocation used to apportion leprosy costs from shared costs.

$$Proportion allocation= Proxy leprosy visits\div Number of general OPD$$

| **Sub-centres** | **DNH** | **Umbergaon** |
| --- | --- | --- |
| Sub-centres surveyed | 4 | 4 |
| Mean sub-centres per PHC | 6 | 9 |
| DNH Building (Avg. area sq. ft.) | 320 | 320 |
| Equipment | Basic GP chamber set-up | |
| Overheads | Electricity and telephone collected. Rest covered under PHC costing | |
| Medicine and consumables | Covered under PHC costing | |
| Human resource | Covered under PHC HR costing | |

**Additional file Table 5. Sub-center standard case: resource parameters**

GP: General practitioner

Basic GP chamber set-up: Examination table, Table, Chairs, Fan, Scales (weight and height), Locked cabinet, Stethoscope, Blood pressure meter (sphygmomanometers), Thermometers, Otoscopes, Tongue depressors, Penlights

**Additional file Table 6. LPEP annual implementation performance in DNH 2015-16 (all PHCs)**

| **LPEP Coverage** | **Number** |
| --- | --- |
| Number of Index Cases | 820 |
| Number of Contacts | 18,094 |
| Number of Contacts listed per Index Case | 22 |
| Number of Contacts screened | 18,031 |
| Contacts suspected of leprosy | 106 |
| Contact given SDR | 14,077 |
| New cases detected through screening | 23 |

| **Additional file Table 7. Annual cost categorization in DNH and Umbergaon**   \| **Type of cost** \| **DNH (n=4)** \| \| \| **Umbergaon (n=4)** \| \| \| \| --- \| --- \| --- \| --- \| --- \| --- \| --- \| \| **Mean INR** \| **USD** \| **%** \| **Mean INR** \| **USD** \| **%** \| \| **Fixed cost** \| 52,355 \| 781.4 \| 2.7 \| 16,125 \| 240.7 \| 2.2 \| \| **Variable** \| 1,895,486 \| 28,290.8 \| 97.3 \| 726,365 \| 10,841.3 \| 97.8 \| \| **Total** \| 1,947,841 \| 29,072.3 \| 100.0 \| 742,490 \| 11,081.9 \| 100.0 \| |
| --- | --- | --- | --- | --- | --- | --- | --- | --- | --- | --- | --- | --- | --- | --- | --- | --- | --- | --- | --- | --- | --- | --- | --- | --- | --- | --- | --- | --- | --- | --- | --- | --- | --- | --- |
